# Thai stakeholders’ awareness and perceptions of the patient adverse event reporting system for herbal medicines: a qualitative study

**DOI:** 10.1007/s11096-022-01533-1

**Published:** 2023-02-06

**Authors:** Wiwan Worakunphanich, Wimon Suwankesawong, Sitaporn Youngkong, Montarat Thavorncharoensap, Claire Anderson, Li Shean Toh

**Affiliations:** 1 Doctor of Philosophy Program in Social, Economic, and Administrative Pharmacy, Department of Pharmacy, Faculty of Pharmacy, Mahidol University, Bangkok, Thailand; 2 Department of Thai Traditional and Alternative Medicine, Thai Traditional Medicine Research Institute, Ministry of Public Health, Nonthaburi, Thailand; 3 The College of Pharmaceutical and Health Consumer Protection of Thailand, Bangkok, Thailand; 4 Health Technology Assessment Graduate Program, Mahidol University, Bangkok, Thailand; 5 Social and Administrative Pharmacy Excellence Research (SAPER) Unit, Department of Pharmacy, Faculty of Pharmacy, Mahidol University, Bangkok, Thailand; 6 Division of Pharmacy Practice and Policy, School of Pharmacy, University of Nottingham, Nottingham, NG7 2RD UK

**Keywords:** Herbal medicine, Patient adverse event reporting system, Pharmacovigilance, Safety monitoring system

## Abstract

**Background:**

In Thailand, the consumption of herbal medicines has been increasing. Adverse events (AEs) of herbal medicines have been identified through the spontaneous reporting system. However, the number of patients reporting AEs of herbal medicines remains limited.

**Aim:**

To explore the awareness and perceptions about the patient reporting system and to explore attitudes towards safety of herbal medicines, experiences, and intention to report AEs of herbal medicines.

**Method:**

Semi-structured in-depth interviews were conducted with stakeholders (patients, community pharmacists, village health volunteers, and consumers who had experienced submitting a complaint about health products to the Consumers Foundation). Additionally, a focus group discussion was held with stakeholders (academics, herbal medicine manufacturers, healthcare professionals, policy maker who was responsible for promoting the use of herbal medicines, pharmacovigilance staff, patient, and representative from patient organisations). The data were audio recorded, transcribed verbatim and analysed using thematic analysis.

**Results:**

Fifty participants were interviewed and the focus group discussion included 12 participants. Patients had positive attitudes towards the safety of herbal medicines. Lack of awareness of the patient reporting system was identified. Nevertheless, all stakeholders acknowledged the importance of the safety monitoring of herbal medicines and indicated a willingness to report AEs via the patient reporting system in the future. A simple reporting system, a variety of reporting channels, the provision of feedback, and providing rewards would motivate patients to report AEs.

**Conclusion:**

Although there is a lack of awareness, this provides a great opportunity to improve patient AE reporting system for herbal medicines in Thailand.

**Supplementary Information:**

The online version contains supplementary material available at 10.1007/s11096-022-01533-1.

## Impact statements

- Lack of awareness of the patient reporting system was identified among Thai stakeholders. Nevertheless, all stakeholders were willing to be involved in the patient AE reporting system.
- A variety of channels for the patient reporting system, such as a mobile application, social media or reporting through Thai traditional medicine doctors and village health volunteers, will facilitate patients to report AEs.
- Simple reporting form, provision of feedback, and providing rewards to express appreciation could motivate Thai people to report AEs.

## Introduction

Herbal medicine is an important part of health care [1]. Currently, many herbal medicinal products are available over-the-counter and can be self-prescribed without consulting a healthcare professional (HCP) [2]. Given the global growth in use of herbal medicines [3], and the growing concerns about their safety, pharmacovigilance systems for herbal medicines are becoming increasingly important [4]. Like other medicines, spontaneous reports are currently used as the main method to identify signal detection of adverse events (AEs) from herbal medicines [5–9].

Patient or consumer reports are report of suspected AEs related to medicinal products as initiated by the patient or consumer without interpretation by a HCP [10]. To date, several countries incorporate direct patient AEs reporting into their pharmacovigilance systems. Patients reporting of AEs is recognised as an important source of information in identifying signals of unknown effects of non-prescription medicines including herbal medicines [4, 10]. In addition, patient reporting could solve the problem of under-reporting among HCPs [10].

In Thailand, the use of herbal medicines is widely promoted by the government. In addition, herbal medicines have been included in the National List of Essential Medicines (NLEM) for reimbursement by the public health insurance system since 1999. The costs of herbal medicine listed in the NLEM prescribed in public hospitals is about 1.2 billion Thai baht (27.5 million GBP) [11].

Since 2010, Thai patients have been able to submit reports of suspected AEs to the Health Product Vigilance Centre (HPVC), under the Thai FDA via postal mail, e-mail, website, and telephone [12]. To date, several severe AEs associated with herbal medicines have been identified through the spontaneous reporting system [5, 13]. Although patient reports are an integral part of monitoring safety of herbal medicines, the number of patient reporting of AEs of herbal medicines in Thailand remains limited. During 2015–2019, spontaneous reports of herbal medicines accounted for less than 10% of the total reports [14], while the number of patient reports in the country accounted for only 0.09% of the total reports [12]. As patient reporting is an important part of the pharmacovigilance system, especially for herbal medicines, it is crucial to increase the number of patient reports.

Besides HCPs, regulatory authorities, pharmaceutical industries, and consumers/patients are considered as key stakeholders in the spontaneous reporting system. It is crucial to understand the awareness and perceptions of all stakeholders regarding patient AEs reporting system as well as their experiences, intention and factors associated with the intention to report AEs in order to develop effective strategies to increase the involvement of patients in the herbal medicines safety monitoring system.

### Aim

To explore the awareness and perceptions about patient reporting system of stakeholders and to explore attitudes towards safety of herbal medicines, experiences and intention to report AEs of herbal medicines among patients in Thailand.

### Ethics approval

Ethical approval was granted by the Institutional Review Board of Mahidol University, Faculty of Dentistry/Faculty of Pharmacy (COA.No.MU-DT/PY-IRB 2020/010.2701) in January 2020.

## Method

### Study design

This qualitative study consisted of semi-structured in-depth interviews and a focus group discussion (FGD). In-depth interviews were conducted to gain detailed information from individuals while FGD offered opportunity for stakeholders to interact and exchange viewpoints. The findings from these 2 methods were used as a complementary and triangulation [15].

### Semi-structured in-depth interviews

#### Study sampling and recruitment

Four groups of participants were included: (1) chronic disease patients, (2) village health volunteers (VHVs). In Thailand, VHVs have been a key part of primary health care. They are people who were selected from the community and were trained by Ministry of Public Health. Responsibilities of VHV include: distribution of health information, public health surveillance, collaboration with the Ministry of Public Health for health promotion activities, and provision of primary health care including first aids for the community, (3) consumers who had experienced submitting a complaint about health products to the Consumers Foundation, and (4) community pharmacists. Inclusion criteria included (1) age > 18 years old, and (2) willing to participate in the study. Participants were recruited using a purposive sampling method. To identify key informants, the research team’s networks (i.e., Thai Patient Society, hospital pharmacists, the Consumers Foundation, and Community Pharmacy Association) were contacted by researcher team. Then, the research team’s networks contacted key informants to seek permission for the researcher (WW) to contact them. If permission was granted, potential participants were contacted and invited to participate in the study. For those who were interested in participating, the interview dates were scheduled. To identify all possible viewpoints, these participants were also selected from a diverse range of characteristics (i.e., gender, age, education, and region of residence) [16].

#### Data collection

After an extensive literature review [17–20], the interview guides were developed and pilot tested in a group of 6 participants, which included pharmacists, academics, pharmacovigilance staff, and qualitative researchers. Interview guides are attached as electronic supplementary material 1. Prior to the start of the interview, written informed consent was obtained from all participants. All interviews were audio recorded. The researcher (WW), who was a PhD student, a pharmacist and worked at Thai traditional and alternative medicine department interviewed the participants in the setting of their choice (e.g., home or workplace). The interviews were conducted between February 2020 to June 2020. It should be noted that thirty in-depth interviews were conducted face-to-face while twenty were telephone interviews due to the COVID-19 pandemic. The in-depth interviews lasted 30–60 min. Data collection was continued until data saturation indicated when the interviews did not yield any new themes [21]. Saturation was reached with varying numbers of each group of participants.

### Focus group discussion

#### Study sampling and recruitment

Participants included academics, herbal medicine manufacturers, a medical doctor, a hospital pharmacist, a community pharmacist, a Thai traditional medicine (TTM) doctor, a policy maker who was responsible for promoting the use of herbal medicines, pharmacovigilance officers, a patient, and a representative from patient organisations. Purposive sampling was adopted. Researcher team identified and invited potential participants via postal mail or e-mail to participate in the FGD.

#### Data collection

Discussion guides were developed and tested by the research team, as shown in electronic supplementary material 1. The focus group discussion was performed at Faculty of Pharmacy, Mahidol University, Thailand in August 2020. Before starting the discussion, the researcher described the objectives of study and all participants provided signed informed consent. They also were informed that the interview would be audio recorded. The discussion lasted for 3 h until data saturation.

### Data analysis

Audio recordings were transcribed verbatim in Thai. Five transcriptions (10%) were checked for accuracy with the audio recordings by WW and one TTM doctor to ensure that the data were trustworthy. Then, Thai transcripts were translated to English. Meaning based translation from Thai to English was performed by WW and 10% of transcripts had forward–backward translations process [22] to check the correctness of the translation by 2 bilingual Thai-English HCPs (one doctor and one TTM doctor). This process was conducted to validate the translations and to maintain the conceptual equivalence [23]. Thematic analysis was used to analyse the interview data by using NVivo qualitative data analysis software (QSR International Pty Ltd., Version 12, 2020) [24]. The analyses of in-depth interviews and focus group discussion were conducted separately. However, the themes which emerged in each group were similar so the findings from the analyses were then combined. In order to ensure reliability of the data analysis, the codes and themes were discussed with the other members of the research team (SY, MT, LST) for agreement [25]. In order to validate the preliminary analyses, major themes were sent back to 10% of each group of participants for them to read and to confirm that the findings accurately reflected their perceptions [26]. Most of participants chose not to respond; only one pharmacist responded who agreed with the findings.

## Results

A total of 50 in-depth interviews were conducted. Characteristics of participants are shown in Table 1. In addition, 12 participants were included in the FDG (i.e., 1 academic, 2 herbal medicine manufacturers, 1 medical doctor, 1 hospital pharmacist, 1 community pharmacist, 1 TTM doctor, 1 policy maker, 2 pharmacovigilance officers, 1 patient, 1 representative from patient organisations).

**Table 1:** Descriptive characteristics of participants in in-depth interview

Participants	Community pharmacists (n = 15)	Village Health volunteers (n = 15)	Consumers who had experienced submitting a complaint about health products (n = 10)	Chronic disease patients (n = 10)
Gender				
Female	9 (60%)	14 (93.33%)	9 (90%)	8 (80%)
Age group (years)				
20–30	1 (6.67%)	0 (0%)	(10%)	0 (0%)
31–40	5 (33.33%)	2 (13.33%)	(40%)	2 (20%)
41–50	2 (13.33%)	5 (33.33%)	(30%)	2 (20%)
51–60	4 (26.67%)	3 (20%)	(20%)	1 (10%)
More than 60	3 (20%)	5 (33.33%)	0 (0%)	5 (50%)
Area of living				
Bangkok	5 (33.33%)	5 (33.33%)	(40%)	8 (80%)
Central region	5 (33.33%)	3 (20%)	(30%)	1 (10%)
North region	0 (0%)	0 (0%)	(20%)	1 (10%)
East region	2 (13.33%)	3 (20%)	(0%)	0 (0%)
North-East region	3 (20%)	4 (26.67%)	(0%)	0 (0%)
South region	0 (0%)	0 (0%)	1 (10%)	0 (0%)
Highest education				
Secondary education/vocational education	0 (0%)	12 (80%)	(20%)	2 (20%)
Bachelor degree	13 (86.67%)	3 (20%)	(70%)	6 (60%)
Higher than bachelor degree	2 (13.33%)	0 (0%)	1 (10%)	2 (20%)

**Fig. 1:**
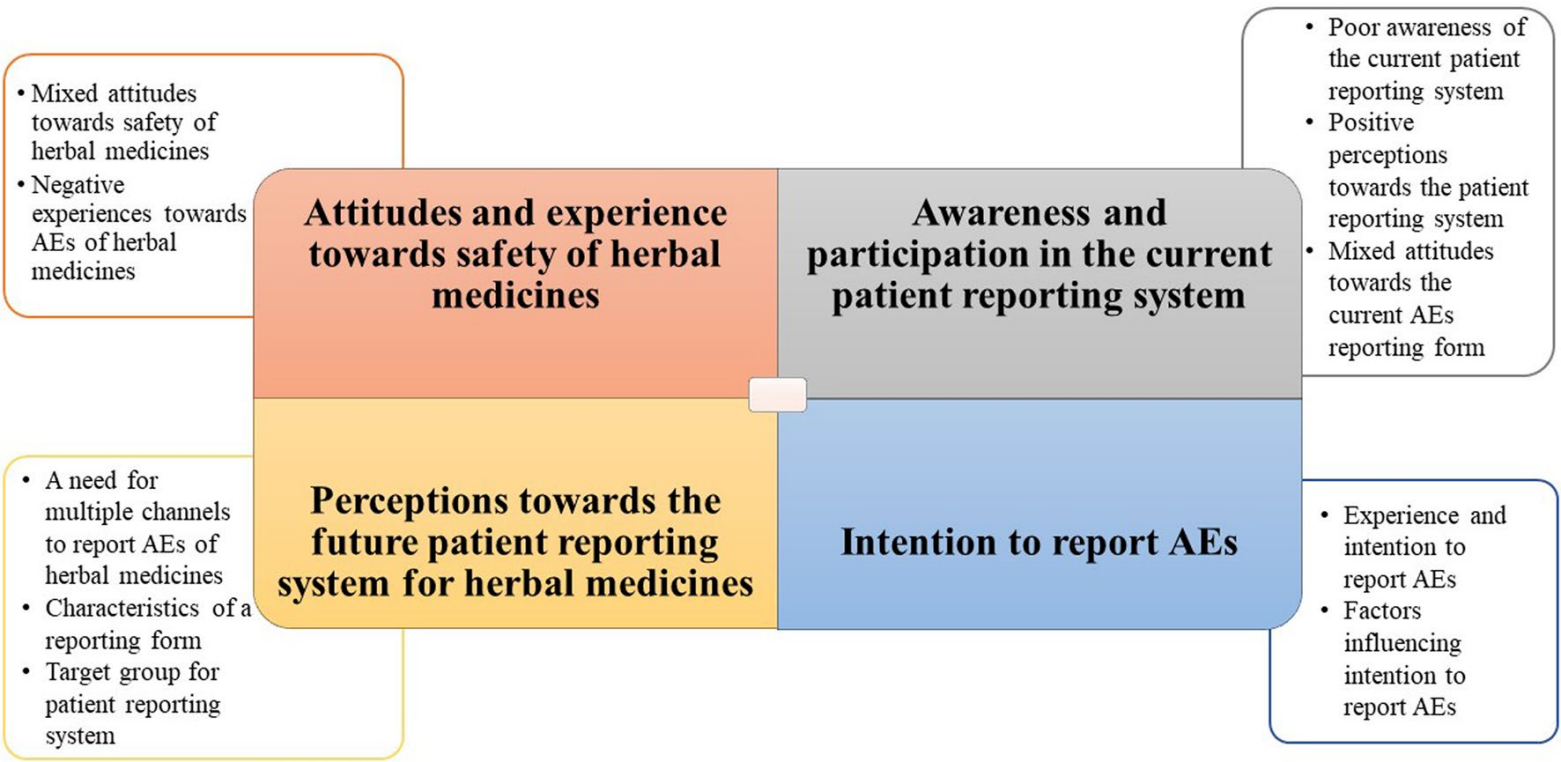
Summary of themes and subthemes

The findings were divided into four major themes as follows: attitudes and experiences towards safety of herbal medicines, awareness and participation in the current patient reporting system, perceptions towards the future patient reporting system for herbal medicines, intention to report AEs, as shown in Fig. 1.

### Theme 1

Attitudes and experiences towards safety of herbal medicines.

### Subtheme 1

Mixed attitudes towards safety of herbal medicines.

Most participants had positive attitudes towards herbal medicines. Many of them mentioned that herbal medicines come from nature and have been used for a long time so that they are safe and effective.

However, some participants, mainly HCPs and those who have previously reported complaints, were concerned about contamination, lack of evidence, and lack of standardization. Additionally, misconceptions of the efficacy of herbal medicines among the general population were identified. The supporting quotes are presented in Table 2.

**Table 2:** Subthemes and supporting quotes in theme 1 and 2

Subthemes	Concepts	Example of supporting quotes
*Theme 1: Attitudes and experiences towards safety of herbal medicines*		
Mixed attitudes towards safety of herbal medicines	Positive attitudes@@Negative attitudes	*“It's good because it comes from nature…and doesn't contain chemical ingredients.” Patient-1 @@ “I'm more concerned about the contamination. Some manufacturing herbal medicines factories may not be well-regulated.” Pharmacist-9*
*Theme 2: Awareness and participation in the current patient reporting system*		
Poor awareness on the current patient reporting system	Poor awareness	*“Patients and pharmacists don’t know about the current system.” Pharmacist-4*
Positive perceptions towards the patient reporting system	The important of patient reporting system	*“…these herbal medicines did not require access through HCPs. This is an important reason why we need patients to be involved.” FGD, Pharmacovigilance officer@@ “It is very important. Information should be gathered from the patients who take medicine. It is important for them to make a report.” VHV-3*
	Validity of data	*“If consumers directly report, there will be a lot of junk information as seen on social media now…The reported information may not be 100% accurate. Someone must verify the data before analysing it.” Complainant-4*
Mixed attitude towards the current AE reporting form	Difficult to fill in reporting form	*“I think the form is too difficult for lay people to understand. It has too much information and consumes too much time to go through.” Complainant-5*

### Subtheme 2

Negative experiences towards AEs of herbal medicines.

AEs of herbal medicines can be severe and cause permanent damage. One participant did not realize that she had experienced AEs from herbal medicine until it became very severe.

### Theme 2

 Awareness and participation in the current patient reporting system.

### Subtheme 1

Poor awareness of the current patient reporting system.

Interestingly, all stakeholders, except the pharmacovigilance officers and a representative from the patient organisations, were unaware of the current patient reporting system. They were not aware that patients could report AEs by themselves. The supporting quotes are presented in Table 2.

### Subtheme 2

Positive perceptions towards the patient reporting system.

Generally, all stakeholders acknowledged that the patient reporting system for herbal medicines was important. As many herbal medicines were available as self-care products, information from patient reporting was therefore useful. Most participants revealed that they were willing to share their AE experiences with others. In addition, they discussed the benefits of patients AE reporting system in that it could contribute to the safety use of herbal medicines and improve the standards of herbal medicines. Nevertheless, some participants were concerned about validity of data from direct patient report. The supporting quotes are presented in Table 2.

### Subtheme 3

Mixed attitudes towards the current AEs reporting form.

Most of the participants were unaware of the current AE reporting form. However, when the researcher showed the current AE reporting form to them, most participants stated that it was quite difficult to fill in as a lot of information was required and the language was difficult to understand. However, a few participants felt they would be able to fill in the current AE reporting form. The supporting quotes are presented in Table 2.

### Theme 3

Perceptions towards the future patient reporting system for herbal medicines.

### Subtheme 1

A need for multiple channels to report AEs of herbal medicines.

Participants suggested that multiple channels of the patient reporting system for herbal medicines should be developed to facilitate patients with variety of capacities and preferences. Most participants considered that patients should be allowed to report via several channels preferably social media, a hotline, a mobile app, and a website. For those who could not report by themselves, e.g., the elderly or who cannot access the internet, they could report through pharmacies, sub-district health promoting hospitals, drug companies, VHVs, and Thai traditional medicine doctors. In addition, most community pharmacists would be willing to be a channel to receive AE reports. Nevertheless, some participants indicated that consumer reports should not go through HCPs. The perception of HCPs that they needed to assess the causality before sending the report might decrease the number of report and reduce the chance of detecting possible AEs. However, some participants suggested that the privacy of system was important. For the channel to access the reporting form, participants suggested several channels which were the same as those channels to report AEs, i.e., a website, a mobile app, social media, pharmacies, and health volunteers. The supporting quotes are presented in Table 3.

**Table 3:** Subthemes and supporting quotes in theme 3

Subthemes	Concepts	Example of supporting quotes
*Theme 3: Perceptions towards the future patient reporting system*		
A need for multiple channels to report AEs of herbal medicines	Multiple channels to report AEs	*“You may have to classify people into 2 levels….Level 1 was the competent group of people, who can report directly. Level 2 was the group of people, who need the mentors to sort things out.” FGD, pharmacovigilance officer*
	Mobile app	*“New generation people would prefer reporting *via* a mobile app.” Pharmacist-6, Female*
	Social media	*“Like now it is an era of mobile phone and internet. It's good to create the channel for people to report *via* Facebook.” Complainant-6*
	Telephone	*“That (hotline) is the channel that everyone can get access to.” VHV-3*
	Online (website)	*“Online channel is the best…it is easy, all people are online now. They usually do online shopping.” Pharmacist-14*
	Community pharmacist	*“I think that it is the pharmacists’ role. Because they bought medicine from us, they usually return to us if the AE occurred.” Pharmacist-12*
	TTM doctor	*“It's good to report through Thai traditional medicine doctor.…Usually, patients are afraid to tell the (medical) doctors that they were currently taking* herbal medicine*.” VHV-9*
	Village health volunteer	*“Let's start with the village health volunteers because they are very close to the people.” Pharmacist-14*
	Privacy	*“You must ensure that information is not leaked anywhere.” Complainant-4*
Characteristics of a reporting form	Different reporting forms between patients and HCPs	*“It would be ideal if the forms for HCP and lay people could be separated. Simple language should be used for lay people with only the most important information…” Pharmacist-9*
	Upload photo	*“We should be able to easily report the medications’ name or even sending some photos and reporting what AEs we are experiencing.” Complainant-5*
Target group for patient reporting system	Everyone	*“In fact, everyone should be the target group because we cannot distinguish that which group will have more AEs than which group.” Pharmacist-4*

### Subtheme 2

Characteristics of a reporting form.

Participants suggested that patients and HCPs should have different reporting forms. The patient reporting form should be simple, e.g., using tick boxes and a function that enabled photos to be uploaded. In addition, the language should be easy to understand. However, the reporting form for herbal medicines and western medicines could be the same. The supporting quotes are presented in Table 3.

### Subtheme 3

Target group for patient reporting system.

Participants suggested that everyone should contribute to the patient reporting system but it should be focused on elderly and patients with chronic disease, who were more likely to develop AEs. The supporting quotes are presented in Table 3.

### Theme 4

Intention to report AEs.

### Subtheme 1

Experience and intention to report AEs.

Although almost all participants had never reported AEs by themselves, as they were unaware of the patient reporting system, most were willing to report AEs in the future. The supporting quotes are presented in Table 4.

**Table 4:** Subthemes and supporting quotes in theme 4

Subthemes	Concepts	Example of supporting quotes
*Theme 4: Intention to report AEs*		
Experience and intention to report AEs	Willing to report AEs	*“Certainly yes…This information can be used for research in the future. Later on, this information (AEs) could be put on the box of herbal medicines.” Patient-2*
Factors influencing intention to report AEs	Characteristics of reporting system	*“I’d rather not report if it's complicated to fill in or submit or there is too much information to read. Thai people definitely don't like it.” Patient-9*
	Feedback	*“The best thing to do and be useful is to follow up and get back to the person who reported it.” Complainant-7*
	Positive impact	*“Thai people, in particular, love helping others. When we see people in need for help, we will help or donate quickly…So I think more people would be interested if we make them realized the benefits from doing this (report AE).” Pharmacist-7*
	Providing reward	*“You might use a scoring system to attract them. Then, you can use the score in exchange for something such as sticker in Line application. Just like when you donate blood, you will receive a score and end up with a medal.” VHV-3*
	Awareness of reporting system	*“People do not understand the importance of reporting. Some people just did not report because they did not think it is important.” Pharmacist-12*
	Lack of knowledge	*“When something bad happens, people want to complain anyway…For Thais, if they know that they can report they would love to report. However, the problem is that they don’t know how to report.” Complainant-3*
	Promotion via social media	*“At present, social media is popular for everyone. But it depends on the public relations unit how they do marketing activity.” VHV-5*
	Promotion via patient organisation	*“If we want them (patient) to play a role, we have to strengthen them…. And when they are strong enough, they will report a lot.” FGD, Representative from patient organisation*
	Promotion via pharmacy	*“These people usually go to the pharmacy where they purchase the products. Running the campaign or informing through community pharmacist can be another strategy.” Pharmacist-12*
	Promotion via village health volunteer	*“It needs to be the villages' or sub-districts' public relations. Because they know the community well and they speak the same language.” Pharmacist-3*
	Promotion via influencer	*“Super Stars or famous people can help promote it (patient reporting system). Because they have a lot of fan clubs, ten million or five million followers.” Complainant-8*

### Subtheme 2

Factors influencing intention to report AEs.

Factors contributing to the intention to report/not report included the ease of the reporting system, the provision of feedback, the impact of the report, the severity of AEs, awareness of reporting systems and its importance, and promotional strategies.

Most participants mentioned that the characteristics of reporting system were important factors that would influence their willingness to report AEs. Participants mentioned that if the reporting system was difficult to access, they would not want to report. Some suggested that the feedback or acknowledgement of the report was the motivation to report and that a summary of the reporting data should be provided to the reporter.

Participants mentioned that impact of the report was a factor affecting their intention to report. Positive impact, e.g., sharing experience to help others, providing rewards, and contributing to the safety data of product, would motivate people to report AEs. However, negative impacts, e.g., privacy violation of the reporter, were an obstacle to report AEs. The severity of symptoms also contributed to their intention to report AEs. Some participants stated that they might not report if the symptoms were mild.

Participants indicated that awareness of the reporting system and its importance affected their intention to report. Lack of knowledge about the products or uncertainty about causal-effect also contributed to unwillingness to report. To increase the number of reports, information relating to the patient reporting system for herbal medicines should be provided. Promotional strategies should also be implemented to raise public awareness. These includes advertising via media and social media, community pharmacists, patient organisations, health volunteers, and influencers. The supporting quotes are presented in Table 4.

## Discussion

### Statement of key findings

While the use of herbal medicines in Thailand has increased, most of the general public were unaware of the AEs of herbal medicines. In addition, this study found that most of participants were unaware of the patient AE reporting system including the existence of the reporting forms. Nevertheless, our study found that all stakeholder acknowledged the importance of a patient reporting system and expressed their interest to monitor the safety of herbal medicines.

Several strategies to improve the patient AE reporting system were identified. These include (1) the development of various channels for patients to report AEs, especially a mobile app or social media, (2) adaptation of reporting form to be user-friendly, (3) provision of feedback, (4) providing rewards, and (5) increase awareness of the patient reporting system. Also, participants preferred to have community pharmacists, VHVs, and traditional medicine doctors to be included in the AE reporting of herbal medicines.

### Interpretation

This study shows that most participants had a positive attitude towards AEs of herbal medicines. Consistent with previous studies [27–29], there was a misconception among Thai people that herbal medicines are safe and carry no risk. Similar to previous studies [29, 30], pharmacists in our study expressed that herbal medicines might not be safe and that contamination might cause AEs [4]. This study found that most of participants were unaware of the patient reporting system. Similar to other countries [31–33], most patients and HCPs had low awareness of the patient reporting system. This could contribute to the very low number of patient reports received in Thailand. Consistent with a previous study, lack of awareness is an important factor for under-reporting [34]. However, most participants had positive attitude towards patient reporting of AEs which is consistent with a previous study [35].

While Thai patients can report via postal mail, e-mail, website, and telephone [12], several channels for patients to report AEs were suggested. This is consistent with the WHO guidelines, which recommend that the means of reporting for the general public should be as simple as possible [10]. We observed that the most of the participants were in favour of online reporting via a website and social media. This could be explained by the fact that the number of internet user among Thai people is recently increasing and social media has become more popular in the country. In 2020, more than 75% used the internet and they spent around 10 h on a daily basis using the internet [36]. In addition, social media penetration in Thailand was 81.2% [37]. Consistent with a recent study, there was a positive attitude toward reporting AEs via social media [38]. According to our study, a mobile app would also be a popular reporting channel and was suggested by most participants. This is in line with patients in other countries, e.g., the UK [39] and India [40]. In India, the percentage of reports increased by 96.45% within 1 year after the mobile app was launched [40]. Moreover, the number of mobile phone users in Thailand reached around 76.58% in 2020 and the most used device for surfing the internet was a mobile phone [41]. Therefore, a mobile app for reporting AEs among Thai people should be developed and assessed. Patients can now report AEs through a pharmacist or a doctor. Consistent with the findings, participants preferred that pharmacists should be included in the system for AE reporting of herbal medicines. This is due to the fact that most herbal medicine are supplied from pharmacy and traditional medicine doctors. This study indicates that some participants felt scared to inform their doctor about their use of herbal medicines. Similar to a previous study, herbal medicine users were less likely to consult their doctor for AEs related to herbal medicines [42]. A previous study indicated that Thai traditional medicine doctors, who can prescribe and dispense herbal medicine in hospital, agreed that AE reporting related to herbal medicines was important and that could improve the safety of herbal medicines [30]. Thus, Thai traditional medicine doctors should be engaged in the safety monitoring of herbal medicines. Moreover, some participants suggested new channels to report using other avenues, such as health volunteers. In Thailand, village health volunteers make a significant contribution to public health in the community [43]. Despite the fact that most village health volunteers in this study were unaware of the patient reporting system, they were willing to help patients to report AEs.

Currently, the paper reporting form for patients and HCPs is the same in Thailand [12]. The findings suggested that the form for patient reports should be different from the HCP report. A new paper reporting form, which is easy to fill out, was requested. Consistent with the WHO guideline [10], patient and HCP forms should be different, in that the form for patients has to be understandable and use layperson’s language and should also include patient-specific questions.

Our study identified that the provision of feedback would motivate patient to report AEs. This is similar to previous studies that patients would like to receive feedback after submitting a report [19, 44]. Nevertheless, there is no the provision of feedback to reporter in Thailand [12]. Additionally, altruistic reasons, e.g., sharing AE experience to prevent others suffering, were also a motivation for patients to report AEs. Altruistic attitudes are a part of Thai culture [45]. Similar to other studies [46, 47], altruistic views encouraged patients to report AEs. Our study indicated that there was lack of awareness and inadequate knowledge, consistent with previous studies [19, 29, 48]. Thus, strategies are needed to increase public knowledge and awareness of the availability, importance, and process of the patient reporting system. Currently, there is a lack of promotional activity to raise the awareness of the general public in Thailand [12]. According to previous studies, a promotional campaign could be conducted in the pharmacy or via patient organisations. This is consistent with previous reviews that countries with high reporting rates (i.e., Denmark, the Netherlands, and the UK) promoted AE reporting among patients via the patient organisations [12]. To improve patient reporting systems, promotional activity should be implemented in Thailand.

### Strengths and weaknesses

Our strengths derived from the comprehensive types of stakeholders in the interviews and FGD. In addition, triangulated data were collected from both in-depth interview and focus group discussion to ensure credibility of the findings. Nevertheless, transferability of the findings should be made with caution as the participants were purposively selected from particular areas in Thailand.

### Further research

A mobile application reporting channel was suggested as a way forward. A further study on the values and suitable features associated with patients reporting via a mobile app should be explored. There was also a positive attitude toward reporting AEs via social media [38]. Further studies on the feasibility of implementing social media as a channel to report AEs should be explored.

## Conclusion

Although lack of awareness regarding patient reporting system was identified, the involvement of patients in the AE reporting system for herbal medicines was viewed as important by Thai stakeholders. Strategies to improve the patient reporting system of AEs of herbal medicines in Thailand include the development of new and various channels to report AEs, especially via a mobile application, as well as the development of simple reporting system. Furthermore, the provision of feedback, providing rewards, promotional interventions towards the patient reporting systems should be implemented.

## Supplementary Information

Below is the link to the electronic supplementary material.

Supplementary file1 (DOCX 16 KB)
