# Supplementary material for: Thai stakeholders’ awareness and perceptions of the patient adverse event reporting system for herbal medicines: a qualitative study
Source: Int J Clin Pharm. 2023 Feb 6;45(2):491–501. doi: 10.1007/s11096-022-01533-1 (PMC9901401; doi:10.1007/s11096-022-01533-1)
Supplement: Supplementary file 1 — Supplementary file1 (DOCX 16 KB) [file 11096_2022_1533_MOESM1_ESM.docx]

**Electronic supplementary material 1**

**Topic guide for in-depth interview**

1. General information of participants (age, gender, education, area of living, occupation)
2. Attitude and experiences regarding the safety of herbal medicine
   1. What do you thing about the safety of herbal medicine?
   2. Have you ever experienced an AE from herbal medicine?
3. Awareness towards the current AE reporting by patient system in Thailand (Channel, method, reporting form)
   1. Have you ever heard that patient can report AEs? If so, do you know the channel and method to report?
   2. What do you think about current reporting form? (show the current reporting form to participant)
4. Perceptions towards the current AE reporting system for herbal medicines
   1. What do you think about the current AE reporting system for herbal medicine?
   2. In your opinion, is patient reporting system important?
5. Experiences and intention to report AE of herbal medicines
   1. Have you ever involved in patient reporting of AE related to herbal medicines?
   2. If you have an AE from herbal medicine in the future, do you willing to report? Why? Why not?
      1. What is your motivation for reporting AE or supporting patient to report AE?
      2. What are the obstacles for patients to report AE?
      3. What is your expectation after reporting?
6. Perceptions towards the appropriate characteristics of patient reporting system for herbal medicines in the future
   1. Which method that you think is appropriate for AE reporting by patient? And Why?
   2. Which reporting form that you think is appropriate for AE reporting by patient? And Why?
   3. Where to access reporting form by patient?
   4. What could be done to promote patient report AE?
   5. What could be done to increase the involvement of patients into the safety monitoring system for herbal medicine?
   6. Other strategies to increase the involvement of patients to report AEs

**Topic guide for focus group discussion**

1. What do you think about the current role of patients in the safety monitoring system for herbal medicines in Thailand?
2. What do you think about the current patient AE reporting systems for herbal medicine in Thailand? Please suggest the appropriate characteristics of patients reporting of AE for herbal medicines in the future? (e.g., channel to retrieve and submit the report, reporting form, etc.)
3. What should be the role/responsibility of each stakeholder in supporting AE reporting by patient?
4. What could be done in the future to promote patient’s involvement and increase patients’ awareness related to AEs reporting?
5. Is there anything you would like to suggest for improve the safety monitoring system for herbal medicines in Thailand?
6. Which group of patients should be the initial target for promoting the involvement in safety monitoring system for herbal medicines? And Why?
